# Therapy in Patients with Hematologic Malignancies, Seroconversion Rates, and SARS-CoV-2 Vaccination

**DOI:** 10.1093/oncolo/oyac169

**Published:** 2022-08-26

**Authors:** Won Sriwijitalai, Viroj Wiwanitkit

**Affiliations:** Private Academic Consultant, Dimapur, India; Honorary Professor, Dr DY Patil University, Pune, India

## Abstract

This letter to the editor comments on a recently published article on seroconversion rates after COVID-19 vaccination.

We would like to share ideas on the publication “Impact of Therapy in Patients with Hematologic Malignancies on Seroconversion Rates After SARS-CoV-2 Vaccination”.^1^ Guven et al found that seroconversion rates following SARS-CoV-2 vaccination were considerably lower in patients with hematologic malignancies, particularly in patients with chronic lymphocytic leukemia and patients treated with anti-CD20 antibodies or BTKi, than in healthy control subjects.^1^ We agree that a vaccination recipient, with a malignancy, receiving concomitant cancer therapy may experience a change in immunogenicity. This reported observation on immunological response should be carefully considered. On the other hand, COVID-19 that is asymptomatic should be diagnosed because it is a prevalent clinical condition.^2^ Guven et al^1^ approaches are insufficient to rule out asymptomatic COVID-19 before or after vaccination. As a result, the conclusions of the current paper must be viewed with caution.
